# Phylogenetic comparison of exonic US4, US7 and UL44 regions of clinical herpes simplex virus type 1 isolates showed lack of association between their anatomic sites of infection and genotypic/sub genotypic classification

**DOI:** 10.1186/1743-422X-9-65

**Published:** 2012-03-14

**Authors:** Anusha Harishankar, Malathi Jambulingam, Raajaram Gowrishankar, Annapoorni Venkatachalam, Umashankar Vetrivel, Sathyabaarathi Ravichandran, Samson Moses Yesupadam, Hajib Narahari Rao Madhavan

**Affiliations:** 1 Larsen & Toubro Microbiology Research Centre, Sankara Nethralaya, No.18, College Road, Chennai 600006, India; 2 SASTRA University, Tirumalaisamudram, Thanjavur 61340, Tamil Nadu, India; 3 Department of Bioinformatics, Sankara Nethralaya, No.18, College Road, Chennai 600006, India

## Abstract

**Background:**

HSV-1 genome is a mosaic of recombinants. Clinical Herpes simplex virus -1 (HSV1) isolates were already genotyped as A, B and C types based on nucleotide variations at Unique Short (US) 4 (gG) and US 7 (gI) regions through phylogeny. Analysis of Glycoprotein C (gC) exon present on the Unique Long (UL) region had also revealed the existence of different genotypes. Glycoprotein C is mainly involved in initial viral attachment to heparan sulphate on host cell surface facilitating the virus's binding and penetration into cell. As the amount of heparan sulphate on the host cell surface varies according to the cell type, it is plausible that different genotypes bind differentially to cell types. Hence, this study was framed to determine the existence of novel genotypes/sub genotypes in the US or UL regions which could associate with clinical entities.

**Results:**

All the twenty five isolates analyzed in this study were of genotype A as per their gG gene sequences. In case of gI gene, 16 out of 25 were found to be type A and the remaining nine were type B putative intergenic recombinants. Intragenic recombinations were also encountered in both the US genes, with gG possessing novel subgenotypes, arbitrarily designated A1 and A2. The 9 type B isolates of gI genes also branched out into 2 clades due to genetic variations. Glycoprotein C of UL region had two distinct genotypic clades α and β, whose topological distribution was significantly different from that of the US region. Neither the US nor UL regions, however, showed any preference among the genotypes to a specific anatomic site of infection. Even the non synonymous variations identified in the functional domain of gC, were not confined to a particular genotype/clinical entity.

**Conclusion:**

The analyses of the US and UL regions of the HSV-1 genome showed the existence of variegated genotypes in these two regions. In contrary to the documented literature, in which Asian strains were concluded as more conserved than European ones, our study showed the existence of a higher degree of variability among Indian strains. However, the identified novel genotypes and subgenotypes were not found associated with clinical entities.

## Background

Herpes simplex virus -1 commonly causes superficial watery blisters in humans in the oral mucosa or genitalia. Apart from infecting the dermis and muco-cutaneous regions, the virus is also capable of infecting a wider range of host tissues, especially of neuronal and corneal origin, leading to encephalitis and keratitis with high rates of morbidity and mortality [1-5].

It is imperative that nuances of replication of HSV are well understood in order to discern the reason behind the wide spectrum of tissues infected. Among the many glycoproteins and glycosaminoglycans adorning a host cell surface, it has been conclusively proved that heparan sulphate (HS), ubiquitously expressed on various cell surfaces, plays an important role in the viral attachment and penetration. HSV-1 penetration and membrane fusion with host cell surface HS takes place via viral glycoproteins C, B, H, L and D [6-8].

HSV-1 contains several glycoproteins, each with varied functions, concerning the overall pathogenesis and immune evasion by the virus. Glycoprotein C (gC) plays a significant role in the efficient attachment to the cell surface [9-11], and Glycoprotein G (gG)interacts with host immune system effecting successful evasion by the virus [12-14]. Glycoprotein I (gI) forms a hetero-dimeric complex with glycoprotein E and is responsible for cell to cell viral spread in epithelial and neuronal cells [15].

A detailed study of the molecular evolution of glycoprotein genes G and I, in European strains, threw up existence of genotypes arbitrarily labeled A, B, C and intragenic recombinants in a hitherto considered stable genetic make up [16]. Subsequent genomic studies carried out by Norberg *et al *(2011) [17] also showed HSV -1 to be a mosaic of recombinants. As gC region is essential in the initial binding to the HS moiety, any variations detected in this region would lead to classification of a separate genotype, which may differentially influence the binding to variegated tissues.

Hence the current study was undertaken to chart and compare the phylogenetic pattern of 2 genes (gG and gI) from the Unique Short (US) region and 1 gene (gC) from the Unique Long (UL) region of HSV-1 genome and determine the possibility of veritable association between the clinical sites of infection and genotypes of any or all of the three genes.

## Results and discussion

The entire coding regions of gG (US 4), gI (US7) and gC (UL 44) genes were sequenced for twenty five clinical isolates. The standard strain HSV-1 ATCC 733VR was sequenced in parallel. These sequences were subjected to phylogenetic analyses by Maximum likelihood method using PHYLIP software. Genes targeting glycoproteins G and I were also subjected to RFLP analyses to genotype them as A, B, and C, based on the protocol elucidated by Norberg *et al *(2006), covering smaller intervals within the genes [5].

### RFLP Analysis of gG and gI

The RFLP analyses of both genes showed a bias towards genotype A. All 25 isolates in their gG gene, and 16 out of 25 isolates in their gI gene and in the case of standard strain, both the genes, conformed to type A cleavage pattern. However, the remaining 9 out of 25 isolates in their gI gene were of type B. The varying genotypic pattern observed in these nine isolates indicated the presence of recombination crossovers between the genes and they were designated as putative intergenic recombinants. These recombinants were of variegated specimen origin with 7 being ocular; whilst, the other two were isolated from oral and genital mucosa.

### Sequence and phylogenetic analyses of glycoprotein G

Phylogenetic analysis using Maximum likelihood method was performed for 25 isolates along with standard strain and 37 sequences covering all the three previously classified genotypes from Genbank.

**Figure 1:**
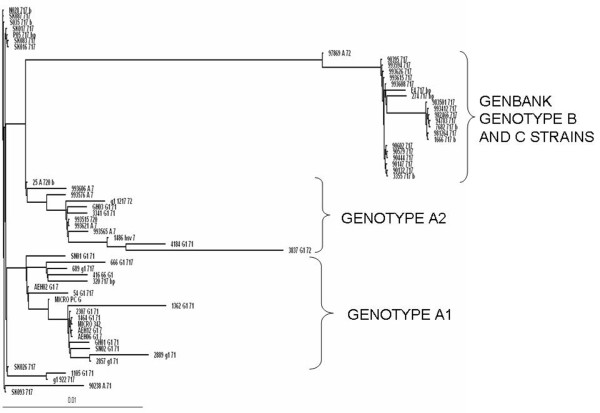
**An unrooted phylogram of gG gene depicting classification of isolates into sub genotypes A1 and A2**.

The phylogenetic tree of gG clearly showed the presence of two distinct genogroups within the isolates. Both the genogroups had considerable evolutionary divergence with respect to Genotype B and C strains, but were very closely related to Genotype A strains obtained from Genbank, in spite of one genogroup being located in the same bifurcating branch, but in a different clade, as B and C strains. RFLP analyses had shown all strains to be Type A. Sequence analyses showed the existence of tandem repeats consisting of 3 nucleotides-GGA repeated nine times, from position 234 to 262 in 6 isolates (with a codon addition seen in 6 isolates), which were subsequently removed prior to phylogenetic analyses. The no. of tandem repeats (n = 9) were characteristic of Type A strains [16]. Hence, divergence into two genogroups indicated the existence of subgenotypes/intragenic recombinants. The groups were arbitrarily labeled as sub genotypes A1 and A2, respectively. An unrooted phylogram of gG gene depicting classification of isolates into sub genotypes A1 and A2 is shown in Figure 1.

An earlier report by Norberg *et al *[16], stated nucleotides T and A at positions 267 & 280, respectively were specific for Genotype A. However, in this study we encountered TG, CG, CC in 22 out of 25 isolates. The other three isolates conformed to the T-A specific genotype A model. These variations were not specific to a particular sub genotype, but as the positions between 280 and 324 were considered to harbour putative recombination crossovers, [16], we provide the proof of variability within the gene, leading to potential recombination occurrence. These variations were seen only among our isolates.

Other variations were seen in the isolates along with a few Genbank isolates at 610, 645 nucleotide positions. Variations in the 267,398, 655 positions were exclusively seen among our strains (genotype A1). These 5 variations were mainly responsible for the subgenotypic classification.

Two ocular isolates did not fall into either of the sub genotype categories.

### Sequence and phylogenetic analyses of glycoprotein I

**Figure 2:**
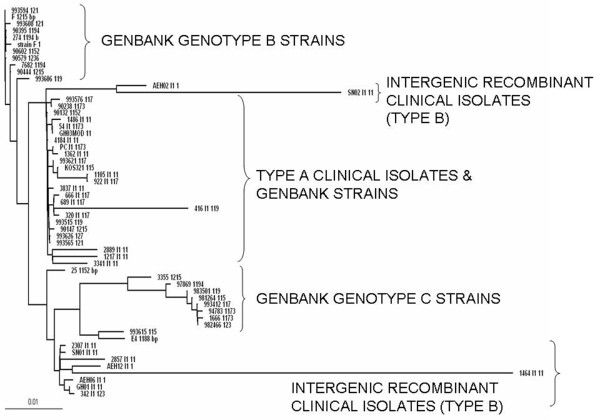
**An unrooted tree of gI gene showing the topological distribution of the isolates**.

Phylogenetic analysis was carried out using Maximum likelihood method for 25 isolates, standard strain along with 31 Genbank strains. A highly divergent tree was obtained. But interestingly, nine strains which were type B as determined by RFLP, and later confirmed by sequencing, did not group themselves together. Seven of them, along with one type A isolate branched out, and exhibited closer relationship to Genbank type C strains. The other two (isolate nos. AEH02 and SN02) were closer to Genbank type B strains. The nine type B isolates flanked the 15 type A clinical isolates and Genbank strains, which were seen grouped together, on either sides. An unrooted tree of gI showing the topological distribution of the isolates is shown in Figure 2.

Variations were seen between positions 270 and 363 in AEH02 and SN02 isolates only, which led them to separate out from other type B isolates. Furthermore, variations at positions 695,698,736,768 in these two isolates were similar to the Genbank Type B and A strains, leading them to be grouped together. The type B isolates which are already intergenic recombinants (comparing gG) showed variations, which could account for presence of putative recombination crossover points within this gene between positions 270 - 363.Though type B isolates had specific variations among them which led their separated cladic topology, no sub genotypic variations were seen among type A isolates.

### Sequence and phylogenetic analyses of glycoprotein C

**Figure 3:**
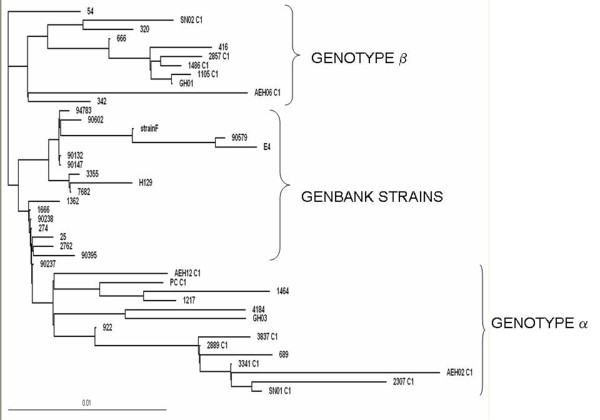
**An unrooted tree of gC gene depicting the existence of geno groups α and β**.

Phylogenetic tree was constructed using Maximum likelihood method by PHYLIP along with 17 Genbank strains. Molecular phylogeny of exonic gC nucleotide sequences threw up existence of only 2 distinct geno groups, though previously 3 separate genogroups were deduced in this region [18]. The genotypes were arbitrarily labeled α andβ, in this region. an unrooted tree of gC gene depicting the existence of geno groups α and β is shown in Figure 3.

The nucleotide variations seen in positions - 381 C- > G,669 G- > A,724 and 725 TA- > AT among typeβ isolates and 781 G- > T among type α isolates were attributed to this cladic separation. Several synonymous and non synonymous substitutions were encountered, but not affecting the formation of α andβ genotypes. Addition of a codon was encountered in 2 corneal isolates (416-ACG and AEH06-ACC) after position 1235.

### Comparison between genotypes and clinical entities

Two novel subgenotypes (A1 and A2) were detected in gG gene, of which type A1 contained all the isolates that turned out to be putative intergenic recombinants on based analyses of gI. These intergenic recombinants, in turn had variations within their gI region, which classified them as intragenic gI type B recombinants. However, the gC gene presented a different picture in terms of topological distribution of isolates, with little correlation between evolutionary pattern seen in the US region. Though there were genotypic variations in all the three genes, none of these classifications were specific to a particular site of infection. The 97 - 367 nucleotide region of glycoprotein C (Amino acid region: 33 - 123) is responsible for its binding to HS moieties on host cell surface, and any mutation in this region will adversely affect its binding capacity [19,20]. In 8 out of 25 isolates studied, we encountered a non synonymous variation at position 187 (A - > C) which resulted in an amino acid change from threonine (polar) to proline (non -polar). Moreover, another variation at position 283(A - > C) in four isolates, resulted in a change from glutamine to lysine. However, none of these changes were specific to a particular genotype or clinical specimen.

The analyses comprised of three genes - gG, gI and gC, of which gG and gI are located in the US region of the genome, close enough to remain linked during replication, whereas, gC present in the UL region is at a considerable genetic distance from the other two genes. The evolutionary patterns of US and UL regions were found to be varied, which can be attributed to the genetic distance between these two regions, accounting for the existence of large number of recombination crossover points. However, surprisingly, despite the proximity of gG and gI genes, even these two genes contained intergenic recombinants and variations in previously characterized potential recombination crossovers [16], thereby making them susceptible to intragenic recombination, which we encountered in both the genes, accounting for the existence of different genotypes as well as sub genotypes. Quantitative evaluation of the genomic polymorphisms of HSV 1 strains from six countries - 3 Asian and 3 non Asian origin (Japan, Korea, China, Sweden, U.S.A. and Kenya) concluded that the evolutionary pattern was similar among same ethnic groups, and the variability encountered among the Asian strains was lesser compared to the Non-Asian ones [21]. Nevertheless, we have seen more variations within our strains (all Indian) with respect to the European strains from Genbank. This in spite of the virus having a stable genome and a low mutation rate of 3.5*10^-8^/site/year [21].

Phylogenetic analysis of 28 European clinical HSV-1 non ocular isolates in their glycoproteins G (gG), I (gI) and E (gE), comprising 2.3% of the unique short (US) region revealed that the sequences could be separated into three genetic groups A, B and C [16].

Despite Duan *et al *[22], mentioning the presence of a type B bias among ocular strains, such a phenomenon was not encountered in the isolates used in our study, comprising of gG, gI and gC genes. All our strains were grouped to either Genotype A or intergenic recombinants. Though novel sub genotypes exist in gG gene, there was no predilection to specific anatomic site. Even the tandem repeats (seen among subgenotype A2 of gG), whose number and nature are more conserved than the other parts of the gene, showed no association with clinical entities. Phylogeny of gC threw up novel genotypes arbitrarily designated as α and β. The gC region is involved in the initial attachment of virus to the host cell. Since different host cells have varying amounts of heparan sulphate on their cell surface, it is possible that a genotypic variation could be anatomic site specific, which was not encountered, making the affinity purely quantitative. Hence, no association was seen between clinical specimen and genotypes on studying three different parts of the HSV genome.

Glycoproteins D, B, H, L apart from gC are involved in the host - virus interactions. Detailed analyses covering all these genes could add on to the input provided by us regarding strain specific site association. However, the high frequency of recombination associated with HSV-1 genome, makes it impossible to assign a particular genotype for a strain [17], sometimes variations are present even in the functional domain, as seen in our study in case of gC. Hence, an increased number of novel genotypes/sub genoytpes due to intergenic/intragenic recombination are likely to be encountered in these glycoprotein genes as well, which will make the segregation of strains to anatomic site difficult.

## Conclusion

Phylogenetic and sequence analyses of clinical HSV-1 isolates comparing three genes from two different segments of the genome revealed the existence of novel genotypes and sub genotypes, adding on the data that widespread variations are present across the genome which is a mosaic of genotypes. As variations seen among our isolates out numbered the variations present in the Genbank strains used in our study, we also differ from the previous study by Sakoaka *et al *[21] that Asian strains are more conserved than their European counterparts by stating that Indian strains have a high degree of variability. However, neither of the two segments of the genome could associate a genotype with a particular clinical entity.

## Methods

### Characteristics of isolates used

A total of twenty five isolates from variegated clinical specimens, 15 ocular, 3 genital, 3 oral, 2 skin and 2 throat swab specimen isolates, were used for this purpose (Table 1). The clinical standard strain employed was HSV1-ATCC 733VR. Apart from these, thirty seven strains for gG [Genbank: AY240815.1, AY240813.1, AY240803.1, AY240755.1, AY240729.1, AY240650.1, AY240818.1, AY240810.1, AY240804.1, AY240741.1, AY240738.1, AJ626499-AJ626526], thirty one sequences for gI [Genbank: AJ626527-AJ626556] and seventeen sequences for gC [Genbank: AJ421502.1, AJ421493.1, AJ421501.1, AJ421507.1, AJ421494.1, AJ421503.1, AJ421491.1, AJ421490.1, AJ421504.1, AJ421497.1, AJ421496.1, GU734771.1, AJ421505.1, AJ421495.1, AJ421492.1, AJ421506.1, and GU734772.1] from Genbank were used as a part of the analyses. The isolates were grown on Vero cell line with Dulbecco's Modified Eagles Medium supplemented with 1% fetal bovine serum and antibiotics.

**Table 1:** Depicts the distribution of clinical entities of various isolates used in the study

S.NO	**ISOLATE NO**.	CLINICAL ENTITY
1	54	Genital Swab
2	320	Genital Swab
3	342	Genital Swab
4	416	Corneal Scraping
5	666	Skin Scraping
6	689	Throat Swab
7	922	Conjunctival Swab
8	1105	Corneal Scraping
9	1217	Throat Swab
10	1362	Corneal Scraping
11	1464	Corneal Scraping
12	1486	Ulcer Swab
13	2857	Corneal Scraping
14	2889	Lip Lesion
15	3341	Corneal Scraping
16	3837	Conjunctival Discharge
17	2307	Corneal Scraping
18	4184	Vitreous Aspirate
19	GH01	Lip Lesion
20	GH03	Ulcer Swab
21	SNO1	Corneal Lesion
22	SN02	Corneal Scraping
23	AEH02	Corneal Scraping
24	AEH06	Corneal Scraping
25	AEH12	Corneal Scraping

### Amplification of gG, gI genes

DNA from isolates and clinical standard strain were extracted using Qiamp DNA mini kit (Germany) according to manufacturer's protocol. The PCR based DNA amplification for gG and gI were done using primers previously documented [16,23].

### Amplification of gC gene

Primers were designed using Primer 3 software to split the exonic gC gene into 5 overlapping sub regions labeled, for convenience, from C1 through C5. The primers are listed in Table 2.

**Table 2:** Depicts the primers designed and employed to amplify gC region

GENE SUB REGION	PRIMER ORIENTATION	PRIMER SEQUENCE (5'-3')
gC1	Forward	CGTGTGGAGGTCGTTTTTCAGT
	Reverse	GTGGTGTTGTTCTTGGGTTTGG
gC2	Forward	AAACCCCAACAATGTCACACAAAAC
	Reverse	CCAAGTAATACATTCCCTGGGTCG
gC3	Forward	GACCCAGGGAATGTATTACT
	Reverse	GTCCTCGAACCAGACAAACT
gC4	Forward	AGTTTGTCTGGTTCGAGGAC
	Reverse	GTCATCGGCAGGTGAAGGTC
gC5	Forward	ACCATCACCATGGAATTTGG
	Reverse	ATGACCTGAGGGGAGAGAGG

The PCR was performed in a 50 μL reaction volume containing 1X PCR buffer with additional 2.5 mM MgCl2 (10 mM Tris with 15 mM MgCl2), 200 mM of each dNTPs, 2.5 units of *Taq *DNA polymerase, 1 mM each primer. The profile consisted of 40 cycles of denaturation at 95°C/30 sec, annealing at 56°C/60 sec for C1, C3-C5 and 62°C/60 sec for C2 and extension at 72°C/60 sec.

The amplicons were gel extracted using Qiagen Gel elution kit and cycle sequenced using Ready reaction mix and analyzed using ABI genetic analyzer AVANT 3130.

### Construction of phylogeny and analyses of tandem repeats

Contig assembly was performed using DNA baser software. Multiple sequence alignment was done using ClustalW2 and Multalin Interface softwares [24]. The nucleotide sequences of gG [Genbank: JN181118-JN181143] and gC [Genbank: JN712694-JN712718] were deposited in Genbank.

Unrooted phylogenetic trees were constructed with maximum likelihood tool with bootstrap validation (500 replicates) using PHYLIP software [25,26]. Tandem repeats were detected using Etandem tool of Emboss package.

Various methods were characterized to serotype and genotype HSV using RFLP [27-29], however, in this paper PCR based RFLP was done using enzymes, targeting 269 bp region within gG and 410 bp region within gI to genotype the isolates [5]. This method was used as it was seen to be applicable to European strains on a larger geographic distribution [22,30].

## Competing interests

The authors declare that they have no competing interests.

## Authors' contributions

MJ conceived the study. HA standardized gC and optimized gI genes PCR, sequenced, analyzed both genes, and drafted the manuscript. RG and AV optimized gG PCR, sequenced and analyzed the gene. UV and SR helped with the Phylogenetic work. SMY was instrumental in data collection. HNM and UV critically reviewed the manuscript. All authors read and approved the final manuscript.

## References

[B1] Furuta Y Fukuda S Chida E Takasu T Ohtani F Inuyama Y Nagashima K Reactivation of herpes simplex virus type 1 in patients with Bell's palsy J Med Virol 1998 54 162 166 10.1002/(SICI)1096-9071(199803)54:3<162::AID-JMV3>3.0.CO;2-3 9515763

[B2] Kimberlin DW Whitley RJ Neonatal herpes: what have we learned Semin Pediatr Infect Dis 2005 16 7 16 10.1053/j.spid.2004.09.006 15685144

[B3] Remeijer L Osterhaus A Verjans G Human herpes simplex virus keratitis: the pathogenesis revisited Ocul Immunol Inflamm 2004 12 255 285 10.1080/092739490500363 15621867

[B4] Whitley R Neonatal herpes simplex virus infection Curr Opin Infect Dis 2004 17 243 246 10.1097/00001432-200406000-00012 15166828

[B5] Norberg P Bergstrom T Liljeqvist J Genotyping of clinical herpes simplex virus type -1 by use of restriction enzymes J Clin Microbiol 2006 44 4511 4514 10.1128/JCM.00421-06 17035491 PMC1698414

[B6] Shieh MT WuDunn D Montgomery RI Esko JD Spear PG Cell surface receptors for herpes simplex virus are heparan sulfate proteoglycans J Cell Biol 1992 116 1273 1281 10.1083/jcb.116.5.1273 1310996 PMC2289355

[B7] Spear P Entry of alphaherpesviruses into cells Semin Virol 1993 4 167 180 10.1006/smvy.1993.1012

[B8] Shukla D Liu J Blaiklock P Shworak NW Bai X Esko JD Cohen GH Eisenberg RJ Rosenberg RD Spear PG A novel role for 3-O-sulfated heparan sulfate in herpes simplex virus 1 entry Cell 1999 99 13 22 10.1016/S0092-8674(00)80058-6 10520990

[B9] Campadelli-Fiume G Stirpe D Boscaro A Avitabile E Foa-Tomasi L Barker D Roizman B Glycoprotein C dependent attachment of herpes simplex virus to susceptible cells leading to productive infection Virol 1990 178 213 222 10.1016/0042-6822(90)90396-9 2167550

[B10] Herold B Spear P Neomycin inhibits glycoprotein C (gC) -dependent binding of HSV-1 to cells and also inhibits postbinding events in entry Virol 1994 203 166 171 10.1006/viro.1994.1469 8030274

[B11] Svennerholm B Jeansson S Vahlne A Lycke E Involvement of glycoprotein C (gC) in adsorption of herpes simplex virus type 1 (HSV-1) to the cell Arch Virol 1991 120 273 279 10.1007/BF01310482 1659800

[B12] Bryant NA Davis-Poynter N Vanderplasschen A Alcami A Glycoprotein G isoforms from some alphaherpesviruses function as broad-spectrum chemokine binding proteins EMBO J 2003 22 833 846 10.1093/emboj/cdg092 12574120 PMC145452

[B13] Rekabdar E Tunback P Liljeqvist J Lindh M Bergstrom T Dichotomy of glycoprotein G gene in herpes simplex virus type 1 isolates J Clin Microbiol 2002 40 3245 3251 10.1128/JCM.40.9.3245-3251.2002 12202560 PMC130675

[B14] Tunback P Bergstrom T Lowhagen G Hoebeke J Liljeqvist J Type-specific reactivity of anti-glycoprotein G antibodies from herpes simplex virus-infected individuals is maintained by single or dual type-specific residues J Gen Virol 2005 86 247 251 10.1099/vir.0.80656-0 15659743

[B15] Dingwell KS Doering LC Johnson DC Glycoproteins E and I facilitate neuron-toneuron spread of herpes simplex virus J Virol 1995 69 7087 7098 7474128 10.1128/jvi.69.11.7087-7098.1995 PMC189628

[B16] Norberg P Bergstrom T Rekabdar E Lindh M Liljeqvist J Phylogenetic analysis of clinical herpes simplex virus type 1 isolates identified three genetic groups and recombinant viruses J Virol 2004 78 10755 10764 10.1128/JVI.78.19.10755-10764.2004 15367642 PMC516408

[B17] Norberg P Tyler S Severini A Whitley R Liljeqvist J-A Bergstrom TA Genome-wide comparative evolutionary analysis of herpes simplex virus type 1 and varicella zoster virus PLoS ONE 2011 6 7 10.1371/journal.pone.0022527 PMC3143153 21799886

[B18] Trybala E Roth A Johansson M Liljeqvist JA Rekabdar E Larm O Bergstrom T Glycosaminoglycan-binding ability is a feature of wild-type strains of herpes simplex virus type 1 Virol 2002 302 413 419 10.1006/viro.2002.1639 12441085

[B19] Grandi P Wang S Schuback D Krasnykh V Spear M Curiel DT Manservigi R Breakefield XO HSV-1 virions engineered for specific binding to cell surface receptors Mol Ther 2004 9 3 419 427 10.1016/j.ymthe.2003.12.010 15006609

[B20] Tal-Singer R Peng C Ponce De Leon M Abrams WR Banfield BW Tufaro F Cohen GH Eisenberg RJ Interaction of herpes simplex virus glycoprotein gC with mammalian cell surface molecules J Virol 1995 69 4471 4483 7769707 10.1128/jvi.69.7.4471-4483.1995 PMC189189

[B21] Sakaoka H Kurita K Lida Y Takada Y Umene K Kim YT Rens CS Nahmias AJ Quantitative analysis of genomic polymorphism of herpes simplex virus type 1 strains from six countries: studies o f molecular evolution and molecular epidemiology of the virus J Gen Virol 1994 75 513 527 10.1099/0022-1317-75-3-513 8126449

[B22] Duan R Van Dun JM Remeijer L Siemerink M Mulder PGH Norberg P Osterhaus ADME Verjans GM Prevalence of herpes simplex virus type 1 glycoprotein G (gG) and gI genotypes in patients with herpetic keratitis Br J Ophthalmol 2008 92 1195 1200 10.1136/bjo.2007.136044 18617539

[B23] Rekabdar E Tunback P Liljeqvist J Bergstrom T Variability of the glycoprotein G gene in clinical isolates of herpes simplex virus type 1 Clin Diagn Lab Immunol 1999 6 826 831 10548571 10.1128/cdli.6.6.826-831.1999 PMC95783

[B24] Corpet F Multiple sequence alignment with hierarchical clustering Nucl Acids Res 1988 6 22 10881 10890 2849754 10.1093/nar/16.22.10881 PMC338945

[B25] Hall TA BioEdit: a user-friendly biological sequence alignment editor and analysis program for Windows 95/98/NT Nucl Acids Symp Ser 1999 41 95 98

[B26] Holmes S Bootstrapping phylogenetic trees: theory and methods Stat Sci 2003 18 241 255 10.1214/ss/1063994979

[B27] Sekine K Molecular-epidemiological and phylogenic analysis of herpes simplex virus type 1 from seven areas in Japan Shika Kiso Igakkai Zasshi 2002 31 5 514 541 2562408 10.2330/joralbiosci1965.31.514

[B28] Umene K Koga C Kame T Discriminant analysis of DNA polymorphisms in herpes simplex virus type 1 strains involved in primary compared to recurrent infections J Virol Methods 2007 139 159 165 10.1016/j.jviromet.2006.09.021 17070937

[B29] Vogel JU Weber B Doerr HW Typing and strain differentiation of clinical herpes simplex virus type 1 and 2 isolates by polymerase chain reaction and subsequent restriction fragment length polymorphism analysis Zentralbl Bakteriol 1994 281 4 502 512 10.1016/S0934-8840(11)80338-5 7727898

[B30] Schmidt-Chanasit J Bialonski A Heinemann P Ulrich RG Günther S Rabenau HF Doerr HW A 10-year molecular survey of herpes simplex virus type 1 in Germany demonstrates a stable and high prevalence of genotypes A and B J Gen Virol 2009 44 513 527 10.1016/j.jcv.2008.12.016 19186100

